# Huntington’s disease among immigrant groups and Swedish-born individuals: a cohort study of all adults 18 years of age and older in Sweden

**DOI:** 10.1007/s10072-021-05085-6

**Published:** 2021-01-30

**Authors:** Per Wändell, Sten Fredrikson, Axel C. Carlsson, Xinjun Li, Jan Sundquist, Kristina Sundquist

**Affiliations:** 1 Division of Family Medicine and Primary Care, Department of Neurobiology, Care Sciences and Society, Karolinska Institutet, Huddinge, Sweden; 2 Division of Neurology, Department of Clinical Neuroscience, Karolinska Institutet Huddinge, Stockholm, Sweden; 3 Academic Primary Health Care Centre, Stockholm Region, Stockholm, Sweden; 4 Center for Primary Health Care Research, Lund University, Malmö, Sweden; 5 Department of Family Medicine and Community Health, Department of Population Health Science and Policy, Icahn School of Medicine at Mount Sinai, New York, NY USA; 6 Center for Community-based Healthcare Research and Education (CoHRE), Department of Functional Pathology, School of Medicine, Shimane University, Matsue, Japan

**Keywords:** Huntington’s disease, Gender, Immigrants, Neighborhood, Socioeconomic status

## Abstract

**Background:**

There is a lack of studies of Huntington’s disease (HD) in immigrants.

**Objective:**

To study the association between country of birth and incident HD in first-generation immigrants versus Swedish-born individuals and in second-generation immigrants versus Swedish-born individuals with Swedish-born parents.

**Methods:**

Study populations included all adults aged 18 years and older in Sweden, i.e., in the first-generation study 6,042,891 individuals with 1034 HD cases and in the second-generation study 4,860,469 individuals with 1001 cases. HD was defined as having at least one registered diagnosis of HD in the National Patient Register. The incidence of HD in different first-generation immigrant groups versus Swedish-born individuals was assessed by Cox regression, expressed as hazard ratios (HRs) and 95% confidence intervals (CI). The models were stratified by sex and adjusted for age, geographical residence in Sweden, educational level, marital status, and neighborhood socioeconomic status.

**Results:**

Mean age-standardized incidence rates per 100,000 person-years were for all Swedish-born 0.82 and for all foreign born 0.53 and for all men 0.73 and for all women 0.81, with the highest incidence rates for the group 80–84 years of age. After adjusting for potential confounders, the HRs were lower in women in the first- and second-generation, i.e., 0.49 (95% CI 0.36–0.67) and 0.63 (95% 0.45–0.87), respectively, and also among women from Finland or with parents from Finland.

**Significance:**

In general, the risk of HD was lower in first-generation and second-generation immigrant women but not among male immigrants.

**Supplementary Information:**

The online version contains supplementary material available at 10.1007/s10072-021-05085-6.

## Introduction

Huntington’s disease (HD) is an autosomal dominant inherited neurodegenerative disease first described in 1872. HD is characterized most of all by progressive motor, cognitive, and psychiatric symptoms [1]. The disease is caused by an expanded CAG repeat within the Huntington’s gene on chromosome 4 [2, 3], with a mean onset of symptoms at around 40 years of age, but with also earlier as well as later onset [1].

There are large differences between different regions of the world, with the lowest recorded prevalence rates of 1 or below per 100,000 in some sub-Saharan African and Asian countries [4]. In Caucasian populations, prevalence rate is estimated to 5.7 per 100,000 in Europe, North America, and Oceania [5] and up to 10 per 100,000 in Western Europe, North America, and Australia [4]. Furthermore, HD is regarded to be more common in people of north European origin [1]. In the UK, the prevalence of identified cases of HD in individuals aged 21 years and above has increased between 1990 and 2010 from 5.4 to 12.3 per 100,000, mostly in older ages [6]. The prevalence rate among men and women in this study was similar, with a mean average prevalence in 1990 and 2010 of men 9.4 and of women 10.4 per 100,000 and with the highest prevalence in the ages 51–60 years [6]. In Canada, the estimated prevalence in 2012 was 13.7/100,000 in the general population and 17.2 in the Caucasian population [7]. Mean age of patients was 56.9 years, mean age of onset of neurological symptoms was 47.9 years, and mean age at diagnosis was 49.7 years.

As regards incidence studies, studies from Europe, North America, and Australia have shown incidence rates between 0.47 and 0.69 per 100,000 [5]. In the UK, the incidence rate was found to be constant between 1990 and 2010 with a rate 0.72 per 100,000 [8]. In Sweden, the annual average incidence rate differs between different regions, with 1.5 per 100,000 in Jämtland in northern Sweden, 0.44 in Region Uppsala in the middle of Eastern Sweden, and 0.59 in the whole of Sweden [9].

These differences by region of origin in the world led us to our aim in the present study, i.e., to estimate the incidence of HD in first- and second-generation immigrants in Sweden compared to Swedish-born individuals and Swedish-born individuals with Swedish-born parents, respectively.

## Methods

### Design

The nationwide registers used in the present study were the Total Population Register and the National Patient Register (NPR). The follow-up period ran from January 1, 1998, until December 31, 2015. A diagnosis of HD from the NPR was registered at the age 18 years and above. Endpoints of the study were thus hospitalization/out-patient care, death, emigration, or the end of the study period on, whichever came first. The NPR includes diagnoses for hospitalized patients, but out-patient diagnoses from specialist care were included from 2001 and onwards. Primary healthcare diagnoses are not included in the NPR. First- and second-generation immigrants were included, divided by region of origin or by region of origin in the parents of the individuals, respectively.

### Outcome variable

Morbus Huntington’s disease (HD) (G10).

### Demographic and socioeconomic variables

The study population was stratified by *sex*.

*Age* was used as a continuous variable in the analysis.

*Educational attainment* was categorized as ≤9 years (partial or complete compulsory schooling), 10–12 years (partial or complete secondary schooling), and >12 years (attendance at college and/or university).

*Marital status* was categorized as married or not being married.

*Geographic region of residence* was included in order to adjust for possible regional differences in hospital admissions and was categorized as (1) large cities, (2) southern Sweden, and (3) northern Sweden. Large cities were defined as municipalities with a population of >200,000 and comprised the three largest cities in Sweden: Stockholm, Gothenburg, and Malmö.

*Region of origin* of first-generation immigrants, or of the parents to second-generation immigrants, was categorized into (1) Finland; (2) Europe (with the exclusion of Finland) and North America; and (3) Asia, Africa, and Latin America.

### Neighborhood deprivation

The neighborhood deprivation index was categorized into four groups: more than one standard deviation (SD) below the mean (low deprivation level or high socioeconomic status (SES)), more than one SD above the mean (high deprivation level or low SES), within one SD of the mean (moderate SES or moderate deprivation level) used as reference group, and also unknown neighborhood SES.

### Statistical analysis

Continuous variables are presented as mean and standard deviations, and categorical variables are presented as counts and percentages. Cox regression analysis was used for estimating the risk (hazard ratios (HR) with 95% confidence intervals (CI)) of incident HD in different immigrant groups compared to the Swedish-born population during the follow-up time. All analyses were stratified by sex. Two models were used in our analyses: Model 1 was adjusted for age and region of residence in Sweden, and Model 2 for age, region of residence in Sweden, educational level, marital status, and neighborhood SES.

We also analyzed age-specific incidence rates for Swedish-born and foreign-born individuals, respectively, and for men and women. Besides, incidence rates over time were analyzed, age-standardized to the European population.

## Results

In the first-generation study (Table 1 and Supplementary Table 1a), 6,042,891 individuals were included (2,902,918 men and 3,139,973 women), with 1034 incident cases (478 men and 556 women), giving a mean age-standardized incidence among men of 0.73 and among women of 0.81 per 100,000 person-years (Supplementary Figure S1). The mean age-standardized incidence among Swedish-born was 0.81 per 100,000 person-years and among foreign-born 0.53 per 100,00 person-years (Supplementary Figure S2). The highest incidence rate among Swedish-born was at 55–59 years of age, while among foreign-born, there were three peaks, i.e., in the years 35–39 years, 55–59 years, and 70–74 years of age, with the highest incidence in the oldest age group.

**Table 1:** The population in first-generation and in the second-generation study and the number of cases of Huntington’s disease

	First-generation individuals				Second-generation individuals			
	Population		Cases		Population		Cases	
	No.	%	No	%	No.	%	No	%
Total population	6042891		1034		4860469		1001	
Gender								
Males	2902918	48.0	478	46.2	2473605	50.9	488	48.8
Females	3139973	52.0	556	53.8	2386864	49.1	513	51.2
Immigrant status*								
Swedish	5040519	83.4	922	89.2	4192944	86.3	902	90.1
Foreign born	1002372	16.6	112	10.8	667525	13.7	99	9.9
Age (years)								
18–39	2225886	36.8	262	25.3	1293899	26.6	173	17.3
40–49	1041269	17.2	239	23.1	1146887	23.6	204	20.4
50–59	1067470	17.7	275	26.6	1042648	21.5	261	26.1
≥ 60	1708266	28.3	258	25.0	1377035	28.3	363	36.3
Educational level								
≤ 9	1955572	32.4	315	30.5	1315776	27.1	275	27.5
10–12	1588380	26.3	340	32.9	1457770	30.0	344	34.4
> 12	2498939	41.4	379	36.7	2086923	42.9	382	38.2
Marital status								
Married	4473576	74.0	798	77.2	2210982	45.5	398	39.8
Not married	1569315	26.0	236	22.8	2649487	54.5	603	60.2
Neighborhood deprivation								
Low	844467	14.0	157	15.2	769956	15.8	157	15.7
Middle	2874189	47.6	494	47.8	2454926	50.5	490	49.0
High	676880	11.2	110	10.6	551242	11.3	101	10.1
Unknown	1647355	27.3	273	26.4	1084345	22.3	253	25.3

*Immigrant status in the second-generation individuals based on the country of birth in parents

In the second-generation study (Table 1 and Supplementary Table 1b), 4,860,469 individuals were included (2,473,605 men and 2,386864 women), with 1001 incident cases (488 men and 513 women). In the first-generation study, there was a similar pattern regarding socioeconomic factors in the population and in HD cases, but among women, less cases had the highest educational level (Supplementary Tables 2a and 2b). In the second-generation study, the pattern was similar in women with foreign-born parents regarding educational level (Supplementary Tables 3a and 3b).

In the first-generation study, among foreign-born men (Table 2), the fully adjusted HR was 0.90 (95% CI 0.68–1.19), and among foreign-born women (Table 2), the fully adjusted HR was 0.49 (95% CI 0.36–0.67), with a significantly lower risk among immigrants from Finland, HR 0.22 (95% CI 0.10–0.49), and Asia/Africa/Latin America, HR 0.44 (95% CI 0.25–0.78).

**Table 2:** Incidence of Huntington’s disease in first-generation male and female immigrants vs Swedish-born men and women, respectively, expressed as hazard ratios (HR) with 95% confidence intervals (95% CI)

		Model 1			Model 2		
	Cases	HR	95% CI		HR	95% CI	
Men							
Born in Sweden	414	1			1		
Born in other countries	64	0.87	0.66	1.13	0.90	0.68	1.19
Finland	10	0.61	0.33	1.14	0.63	0.34	1.19
Other European countries and North America	30	0.87	0.60	1.26	0.91	0.62	1.33
Other regions (Asia, Africa, and Latin America)	24	1.04	0.69	1.58	1.10	0.71	1.70
Women							
Born in Sweden	508	1			1		
Born in other countries	48	**0.55**	**0.41**	**0.73**	**0.49**	**0.36**	**0.67**
Finland	6	**0.25**	**0.11**	**0.55**	**0.22**	**0.10**	**0.49**
Other European countries and North America	29	0.78	0.53	1.13	0.72	0.49	1.05
Other regions (Asia, Africa and Latin America)	13	**0.50**	**0.29**	**0.87**	**0.44**	**0.25**	**0.78**

Model 1: adjusted for birth year, gender, and region of residence in Sweden; Model 2: adjusted for birth year, gender, region of residence in Sweden, educational level, marital status, and neighborhood deprivation

Bold values are statistically significant

In the second-generation study (Table 3), among men with foreign-born parents, the fully adjusted HR was 1.00 (95% CI 0.75–1.32), with a significantly higher risk among men with parents from Asia/Africa/Latin America, fully adjusted HR 2.69 (95% CI 1.56–4.66), and among women with foreign-born parents, the fully adjusted HR was 0.63 (95% CI 0.45–0.87), with a significantly lower risk among women with parents from Finland, fully adjusted HR 0.45 (95% CI 0.25–0.83).

**Table 3:** Incidence of Huntington’s disease in second-generation male and female immigrants vs men and women with Swedish-born parents expressed as hazard ratios (HR) with 95% confidence intervals (95% CI)

		Model 1			Model 2		
	Cases	HR	95% CI		HR	95% CI	
Men							
Parents born in Sweden	429	1			1		
Parents born in other countries	59	1.01	0.77	1.34	1.00	0.75	1.32
Finland	20	0.94	0.60	1.47	0.91	0.58	1.43
Other European countries and North America	25	0.80	0.53	1.20	0.80	0.53	1.20
Other regions (Asia, Africa and Latin America)	14	**2.77**	**1.61**	**4.77**	**2.69**	**1.56**	**4.66**
Women							
Parents born in Sweden	473	1			1		
Parents born in other countries	40	**0.62**	**0.45**	**0.86**	**0.63**	**0.45**	**0.87**
Finland	11	**0.46**	**0.25**	**0.83**	**0.45**	**0.25**	**0.83**
Other European countries and North America	23	0.66	0.44	1.01	0.68	0.44	1.03
Other regions (Asia, Africa and Latin America)	6	1.07	0.48	2.42	1.10	0.49	2.49

Model 1: adjusted for birth year, gender, and region of residence in Sweden; Model 2: adjusted for birth year, gender, region of residence in Sweden, educational level, marital status, and neighborhood deprivation

Bold values are statistically significant

## Discussion

The main findings of this study were significantly lower risks of HD among both first- and second-generation immigrant women in general. Regarding specific groups, women from Finland, and women with parents from Finland, showed especially lower risks. Additionally, a lower risk was found in first-generation women born outside Europe and North America. Higher risks of HD were seen only among second-generation men with parents from outside Europe and North America.

We choose to categorize immigrants into three groups. The prevalence of HD in Finland has been shown to be lower than in other parts of Europe, with a heritage more similar to Asia [10]. Besides, the Finnish group has traditionally been the largest immigrant group in Sweden and is still one of the largest. Interestingly, we only found a lower HD risk in first- and second-generation women but not in men. However, there are also differences within countries, which could be an explanation to the discrepancy between men and women.

European populations, including European descendants in North America and Australia, have a high prevalence of HD [1, 4, 5], and we found no statistically significant differences compared to Swedish-born individuals or Swedish-born individuals with Swedish-born parents.

Regarding the third group, i.e., individuals from Asia, Africa, or Latin America, or with parents from these regions, there are previous studies showing a lower risk of HD. Asian populations are shown to have a very low prevalence [1, 4, 5] but with a prevalence in the Middle East of 3–4 per 100,000 [11], i.e., somewhat lower than in European or Caucasian populations. Studies from Africa and Latin America are scarce but with a low prevalence [12, 13]. However, there are also differences within countries; in Sweden, a higher prevalence has been found in the north-western region [9]. There seems to be an even higher prevalence in some restricted areas in Latin America [12], in Egypt [14], and also in Tasmania [15]. We found a discrepancy between first- and second-generation individuals, with a lower risk in first-generation women, in line with what could be expected, but a higher risk in men with parents from Asia, Africa, and Latin America. We have no explanation to these findings, but the number of cases was rather small.

An overall explanation to a higher prevalence of HD in Swedish-born individuals could be that there is an awareness in Swedish families about the parents and grandparents with HD, potentially increasing the chance of identifying symptoms. Some immigrant groups may hypothetically not have such awareness.

This study has limitations. The number of cases was quite low, why we had to categorize the individuals into a few groups, i.e., Finland, Europe (except Finland), and North America and Asia, Africa, and Latin America. Besides, differences within countries and regions could be substantial. The strengths lie in the Swedish registers that have been shown to be of high quality [16, 17], and we expect that we have detected most cases in the Swedish National Patient Register.

In conclusion, we found a lower risk of HD in first- and second-generation women overall but also in first- and second-generation women from Finland. The findings in men went in a different direction, at least partially. To some part, this higher incidence could be explained by a higher awareness about HD among blood relatives in individuals with Swedish-born parents than in individuals with foreign-born parents.

## Supplementary Information

ESM 1 (DOCX 292 kb).
