# Supplementary material for: Huntington’s disease among immigrant groups and Swedish-born individuals: a cohort study of all adults 18 years of age and older in Sweden
Source: Neurol Sci. 2021 Jan 30;42(9):3851–6. doi: 10.1007/s10072-021-05085-6 (PMC8413185; doi:10.1007/s10072-021-05085-6)

| **Supplementary Table S1a. Population in first-generation study and number of cases of Huntington’s disease events categorized by sex** | | | | | | | | | | | |
| --- | --- | --- | --- | --- | --- | --- | --- | --- | --- | --- | --- |
|  | Men | | | | |  | Women | | | | |
|  | Population | |  | Events | |  | Population | |  | Events | |
|  | No. | % |  | No | % |  | No. | % |  | No | % |
| Total population | 2902918 |  |  | 478 |  |  | 3139973 |  |  | 556 |  |
| Age (years) |  |  |  |  |  |  |  |  |  |  |  |
| 18-39 | 1086756 | 37.4 |  | 116 | 24.3 |  | 1139130 | 36.3 |  | 146 | 26.3 |
| 40-49 | 513920 | 17.7 |  | 101 | 21.1 |  | 527349 | 16.8 |  | 138 | 24.8 |
| 50-59 | 532459 | 18.3 |  | 143 | 29.9 |  | 535011 | 17.0 |  | 132 | 23.7 |
| ≥ 60 | 769783 | 26.5 |  | 118 | 24.7 |  | 938483 | 29.9 |  | 140 | 25.2 |
| Educational level |  |  |  |  |  |  |  |  |  |  |  |
| ≤ 9 | 928587 | 32.0 |  | 144 | 30.1 |  | 1026985 | 32.7 |  | 171 | 30.8 |
| 10-12 | 745586 | 25.7 |  | 138 | 28.9 |  | 842794 | 26.8 |  | 202 | 36.3 |
| > 12 | 1228745 | 42.3 |  | 196 | 41.0 |  | 1270194 | 40.5 |  | 183 | 32.9 |
| Marital status |  |  |  |  |  |  |  |  |  |  |  |
| Married | 1635408 | 56.3 |  | 275 | 57.5 |  | 2838168 | 90.4 |  | 523 | 94.1 |
| Not married | 1267510 | 43.7 |  | 203 | 42.5 |  | 301805 | 9.6 |  | 33 | 5.9 |
| Neighborhood deprivation |  |  |  |  |  |  |  |  |  |  |  |
| Low | 407169 | 14.0 |  | 80 | 16.7 |  | 437298 | 13.9 |  | 77 | 13.8 |
| Middle | 1361540 | 46.9 |  | 219 | 45.8 |  | 1512649 | 48.2 |  | 275 | 49.5 |
| High | 320983 | 11.1 |  | 51 | 10.7 |  | 355897 | 11.3 |  | 59 | 10.6 |
| Unknown | 813226 | 28.0 |  | 128 | 26.8 |  | 834129 | 26.6 |  | 145 | 26.1 |

| **Supplementary Table S1b. Population in second-generation study and number of cases of Huntington’s disease events categorized by sex** | | | | | | | | | | | |
| --- | --- | --- | --- | --- | --- | --- | --- | --- | --- | --- | --- |
|  | Men | | | | |  | Women | | | | |
|  | Population | |  | Events | |  | Population | |  | Events | |
|  | No. | % |  | No | % |  | No. | % |  | No | % |
| Total population | 2473605 |  |  | 488 |  |  | 2386864 |  |  | 513 |  |
| Age (years) |  |  |  |  |  |  |  |  |  |  |  |
| 18-39 | 663984 | 26.8 |  | 89 | 18.2 |  | 629915 | 26.4 |  | 84 | 16.4 |
| 40-49 | 586414 | 23.7 |  | 99 | 20.3 |  | 560473 | 23.5 |  | 105 | 20.5 |
| 50-59 | 531457 | 21.5 |  | 116 | 23.8 |  | 511191 | 21.4 |  | 145 | 28.3 |
| ≥ 60 | 691750 | 28.0 |  | 184 | 37.7 |  | 685285 | 28.7 |  | 179 | 34.9 |
| Educational level |  |  |  |  |  |  |  |  |  |  |  |
| ≤ 9 | 708574 | 28.6 |  | 138 | 28.3 |  | 607202 | 25.4 |  | 137 | 26.7 |
| 10-12 | 708521 | 28.6 |  | 155 | 31.8 |  | 749249 | 31.4 |  | 189 | 36.8 |
| > 12 | 1056510 | 42.7 |  | 195 | 40.0 |  | 1030413 | 43.2 |  | 187 | 36.5 |
| Marital status |  |  |  |  |  |  |  |  |  |  |  |
| Married | 1067475 | 43.2 |  | 191 | 39.1 |  | 1143507 | 47.9 |  | 207 | 40.4 |
| Not married | 1406130 | 56.8 |  | 297 | 60.9 |  | 1243357 | 52.1 |  | 306 | 59.6 |
| Neighborhood deprivation |  |  |  |  |  |  |  |  |  |  |  |
| Low | 386891 | 15.6 |  | 80 | 16.4 |  | 383065 | 16.0 |  | 77 | 15.0 |
| Middle | 1258603 | 50.9 |  | 222 | 45.5 |  | 1196323 | 50.1 |  | 268 | 52.2 |
| High | 286875 | 11.6 |  | 56 | 11.5 |  | 264367 | 11.1 |  | 45 | 8.8 |
| Unknown | 541236 | 21.9 |  | 130 | 26.6 |  | 543109 | 22.8 |  | 123 | 24.0 |

| **Supplementary Table S2a. Population and number of cases of events in male Swedish and first-generation immigrants** | | | | | | | | | | | |
| --- | --- | --- | --- | --- | --- | --- | --- | --- | --- | --- | --- |
|  | Swedish born | | | | |  | Foreign born | | | | |
|  | Population | |  | Events | |  | Population | |  | Events | |
|  | No. | % |  | No | % |  | No. | % |  | No | % |
| Total population | 2403696 |  |  | 414 |  |  | 499222 |  |  | 64 |  |
| Age (years) |  |  |  |  |  |  |  |  |  |  |  |
| 18-39 | 849296 | 35.3 |  | 94 | 22.7 |  | 237460 | 47.6 |  | 22 | 34.4 |
| 40-49 | 416650 | 17.3 |  | 86 | 20.8 |  | 97270 | 19.5 |  | 15 | 23.4 |
| 50-59 | 447600 | 18.6 |  | 128 | 30.9 |  | 84859 | 17.0 |  | 15 | 23.4 |
| ≥ 60 | 690150 | 28.7 |  | 106 | 25.6 |  | 79633 | 16.0 |  | 12 | 18.8 |
| Educational level |  |  |  |  |  |  |  |  |  |  |  |
| ≤ 9 | 730337 | 30.4 |  | 116 | 28.0 |  | 198250 | 39.7 |  | 28 | 43.8 |
| 10-12 | 649863 | 27.0 |  | 121 | 29.2 |  | 95723 | 19.2 |  | 17 | 26.6 |
| > 12 | 1023496 | 42.6 |  | 177 | 42.8 |  | 205249 | 41.1 |  | 19 | 29.7 |
| Marital status |  |  |  |  |  |  |  |  |  |  |  |
| Married | 1252715 | 52.1 |  | 233 | 56.3 |  | 382693 | 76.7 |  | 42 | 65.6 |
| Not married | 1150981 | 47.9 |  | 181 | 43.7 |  | 116529 | 23.3 |  | 22 | 34.4 |
| Neighborhood deprivation |  |  |  |  |  |  |  |  |  |  |  |
| Low | 383925 | 16.0 |  | 75 | 18.1 |  | 23244 | 4.7 |  | 5 | 7.8 |
| Middle | 1266620 | 52.7 |  | 202 | 48.8 |  | 94920 | 19.0 |  | 17 | 26.6 |
| High | 269905 | 11.2 |  | 45 | 10.9 |  | 51078 | 10.2 |  | 6 | 9.4 |
| Unknown | 483246 | 20.1 |  | 92 | 22.2 |  | 329980 | 66.1 |  | 36 | 56.3 |

| **Supplementary Table S2b. Population and number of cases of events in female Swedish and first-generation immigrants** | | | | | | | | | | | |
| --- | --- | --- | --- | --- | --- | --- | --- | --- | --- | --- | --- |
|  | Swedish born | | | | |  | Foreign born | | | | |
|  | Population | |  | Events | |  | Population | |  | Events | |
|  | No. | % |  | No | % |  | No. | % |  | No | % |
| Total population | 2636823 |  |  | 508 |  |  | 503150 |  |  | 48 |  |
| Age (years) |  |  |  |  |  |  |  |  |  |  |  |
| 18-39 | 892929 | 33.9 |  | 132 | 26.0 |  | 246201 | 48.9 |  | 14 | 29.2 |
| 40-49 | 431899 | 16.4 |  | 125 | 24.6 |  | 95450 | 19.0 |  | 13 | 27.1 |
| 50-59 | 461985 | 17.5 |  | 123 | 24.2 |  | 73026 | 14.5 |  | 9 | 18.8 |
| ≥ 60 | 850010 | 32.2 |  | 128 | 25.2 |  | 88473 | 17.6 |  | 12 | 25.0 |
| Educational level |  |  |  |  |  |  |  |  |  |  |  |
| ≤ 9 | 821681 | 31.2 |  | 153 | 30.1 |  | 205304 | 40.8 |  | 18 | 37.5 |
| 10-12 | 744894 | 28.2 |  | 189 | 37.2 |  | 97900 | 19.5 |  | 13 | 27.1 |
| > 12 | 1070248 | 40.6 |  | 166 | 32.7 |  | 199946 | 39.7 |  | 17 | 35.4 |
| Marital status |  |  |  |  |  |  |  |  |  |  |  |
| Married | 2365897 | 89.7 |  | 479 | 94.3 |  | 472271 | 93.9 |  | 44 | 91.7 |
| Not married | 270926 | 10.3 |  | 29 | 5.7 |  | 30879 | 6.1 |  | 4 | 8.3 |
| Neighborhood deprivation |  |  |  |  |  |  |  |  |  |  |  |
| Low | 410405 | 15.6 |  | 74 | 14.6 |  | 26893 | 5.3 |  | 3 | 6.3 |
| Middle | 1406262 | 53.3 |  | 260 | 51.2 |  | 106387 | 21.1 |  | 15 | 31.3 |
| High | 304620 | 11.6 |  | 52 | 10.2 |  | 51277 | 10.2 |  | 7 | 14.6 |
| Unknown | 515536 | 19.6 |  | 122 | 24.0 |  | 318593 | 63.3 |  | 23 | 47.9 |

| **Supplementary Table S3a. Population and number of cases of events in male Swedish and second-generation immigrants** | | | | | | | | | | | |
| --- | --- | --- | --- | --- | --- | --- | --- | --- | --- | --- | --- |
|  | Swedish born | | | | |  | Foreign born | | | | |
|  | Population | |  | Events | |  | Population | |  | Events | |
|  | No. | % |  | No | % |  | No. | % |  | No | % |
| Total population | 2132150 |  |  | 429 |  |  | 341455 |  |  | 59 |  |
| Age (years) |  |  |  |  |  |  |  |  |  |  |  |
| 18-39 | 525778 | 24.7 |  | 67 | 15.6 |  | 138206 | 40.5 |  | 22 | 37.3 |
| 40-49 | 481180 | 20.0 |  | 82 | 19.8 |  | 105234 | 30.8 |  | 17 | 28.8 |
| 50-59 | 466324 | 19.4 |  | 101 | 24.4 |  | 65133 | 19.1 |  | 15 | 25.4 |
| ≥ 60 | 658868 | 27.4 |  | 179 | 43.2 |  | 32882 | 9.6 |  | 5 | 8.5 |
| Educational level |  |  |  |  |  |  |  |  |  |  |  |
| ≤ 9 | 584694 | 24.3 |  | 122 | 29.5 |  | 123880 | 36.3 |  | 16 | 27.1 |
| 10-12 | 620078 | 25.8 |  | 134 | 32.4 |  | 88443 | 25.9 |  | 21 | 35.6 |
| > 12 | 927378 | 38.6 |  | 173 | 41.8 |  | 129132 | 37.8 |  | 22 | 37.3 |
| Marital status |  |  |  |  |  |  |  |  |  |  |  |
| Married | 927678 | 38.6 |  | 177 | 42.8 |  | 139797 | 40.9 |  | 14 | 23.7 |
| Not married | 1204472 | 50.1 |  | 252 | 60.9 |  | 201658 | 59.1 |  | 45 | 76.3 |
| Neighborhood deprivation |  |  |  |  |  |  |  |  |  |  |  |
| Low | 352444 | 14.7 |  | 75 | 18.1 |  | 34447 | 10.1 |  | 5 | 8.5 |
| Middle | 1134313 | 47.2 |  | 204 | 49.3 |  | 124290 | 36.4 |  | 18 | 30.5 |
| High | 241916 | 10.1 |  | 49 | 11.8 |  | 44959 | 13.2 |  | 7 | 11.9 |
| Unknown | 403477 | 16.8 |  | 101 | 24.4 |  | 137759 | 40.3 |  | 29 | 49.2 |

| **Supplementary Table S3b. Population and number of cases of events in female Swedish and second-generation immigrants** | | | | | | | | | | | |
| --- | --- | --- | --- | --- | --- | --- | --- | --- | --- | --- | --- |
|  | Swedish born | | | | |  | Foreign born | | | | |
|  | Population | |  | Events | |  | Population | |  | Events | |
|  | No. | % |  | No | % |  | No. | % |  | No | % |
| Total population | 2060794 |  |  | 473 |  |  | 326070 |  |  | 40 |  |
| Age (years) |  |  |  |  |  |  |  |  |  |  |  |
| 18-39 | 499149 | 24.2 |  | 71 | 15.0 |  | 130766 | 40.1 |  | 13 | 32.5 |
| 40-49 | 459770 | 22.3 |  | 95 | 20.1 |  | 100703 | 30.9 |  | 10 | 25.0 |
| 50-59 | 448716 | 21.8 |  | 131 | 27.7 |  | 62475 | 19.2 |  | 14 | 35.0 |
| ≥ 60 | 653159 | 31.7 |  | 176 | 37.2 |  | 32126 | 9.9 |  | 3 | 7.5 |
| Educational level |  |  |  |  |  |  |  |  |  |  |  |
| ≤ 9 | 492293 | 23.9 |  | 124 | 26.2 |  | 114909 | 35.2 |  | 13 | 32.5 |
| 10-12 | 667061 | 32.4 |  | 179 | 37.8 |  | 82188 | 25.2 |  | 10 | 25.0 |
| > 12 | 901440 | 43.7 |  | 170 | 35.9 |  | 128973 | 39.6 |  | 17 | 42.5 |
| Marital status |  |  |  |  |  |  |  |  |  |  |  |
| Married | 990499 | 48.1 |  | 192 | 40.6 |  | 153008 | 46.9 |  | 15 | 37.5 |
| Not married | 1070295 | 51.9 |  | 281 | 59.4 |  | 173062 | 53.1 |  | 25 | 62.5 |
| Neighborhood deprivation |  |  |  |  |  |  |  |  |  |  |  |
| Low | 349276 | 16.9 |  | 68 | 14.4 |  | 33789 | 10.4 |  | 9 | 22.5 |
| Middle | 1079836 | 52.4 |  | 252 | 53.3 |  | 116487 | 35.7 |  | 16 | 40.0 |
| High | 223003 | 10.8 |  | 42 | 8.9 |  | 41364 | 12.7 |  | 3 | 7.5 |
| Unknown | 408679 | 19.8 |  | 111 | 23.5 |  | 134430 | 41.2 |  | 12 | 30.0 |

Supplementary Figure S1.


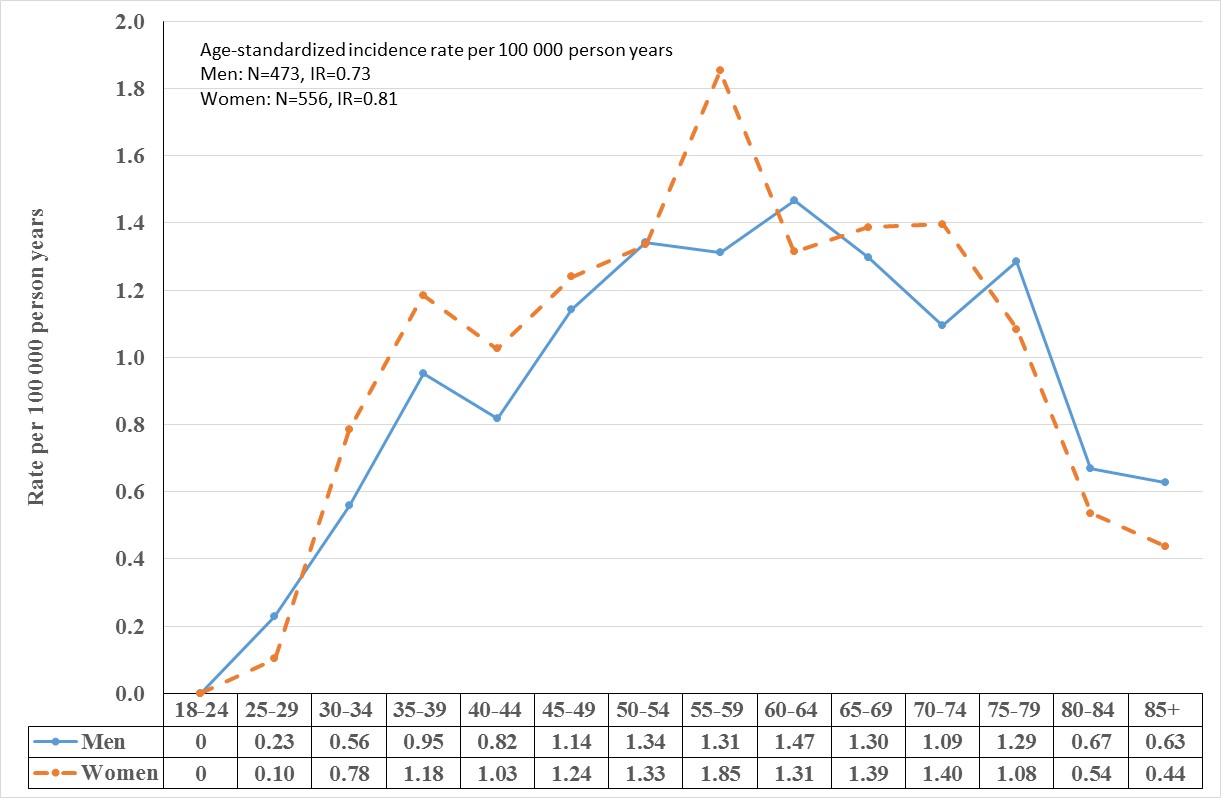


Supplementary Figure S2.


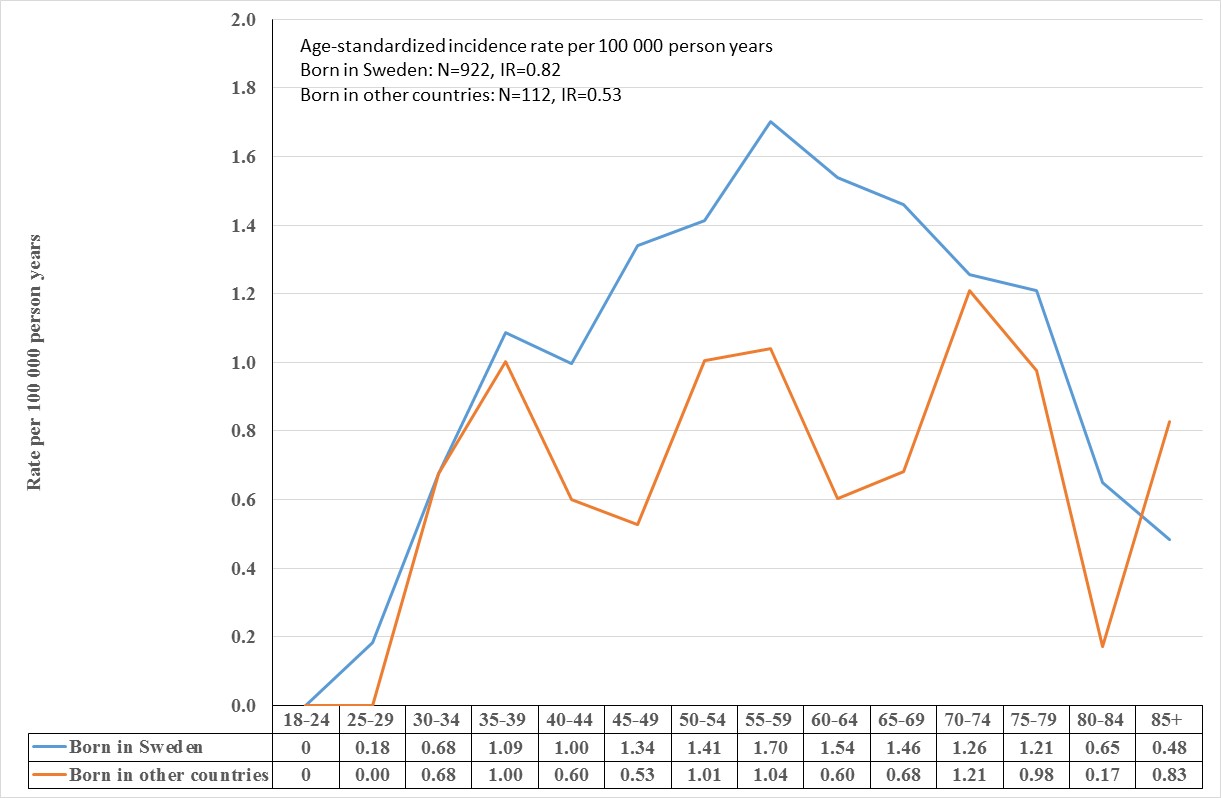

Supplement: Supplementary file 1 — (DOCX 292 kb). [file 10072_2021_5085_MOESM1_ESM.docx]
